# Regression of a symptomatic varix after transarterial embolization of a brain arteriovenous malformation: A case report and literature review

**DOI:** 10.1097/MD.0000000000018418

**Published:** 2019-12-27

**Authors:** Guichen Li, Guangming Wang, Jing Yu, Kun Hou, Jinlu Yu

**Affiliations:** a Department of Neurology; b Department of Neurosurgery; c Department of Operation Room, The First Hospital of Jilin University, Changchun, China.

**Keywords:** brain arteriovenous malformation, regression, transarterial embolization, varix

## Abstract

**Rationale:**

Brain arteriovenous malformation (BAVM)-associated varix is always asymptomatic, and no special treatment is needed. However, there is no consensus regarding how to address a varix that has led to clinical manifestation.

**Patient concerns:**

An 11-year-old girl was admitted complaining of left hemiparesis for 4 days. She was previously healthy and denied any history of similar ictus. She was alert, and a physical examination performed upon admission was unremarkable except for the left hemiparesis.

**Diagnoses:**

Head magnetic resonance imaging (MRI) showed a linear and round flow void and perilesional edema in the region of the right basal ganglia, indicating a BAVM. Gadolinium-enhanced MRI showed peripheral enhancement of the round lesion. Computed tomography angiography (CTA) showed that the BAVM was fed by the ipsilateral posterior cerebral artery and anterior choroidal artery and drained into the vein of Galen. A large varix was also noted at the top of the BAVM and was consistent with the round flow void observed at the right basal ganglia on MRI. The Spetzler-Martin grading scale was grade IV

**Interventions:**

The patient experienced a TAE of the BAVM nidus with liquid embolic agent.

**Outcomes:**

A follow-up investigation showed regression of the varix, although there was still some residual BAVM. The patient experienced a favorable recovery.

**Lessons:**

In the case of a BAVM-associated symptomatic varix, if surgical resection cannot readily be performed, initial TAE of the BAVM nidus can be attempted.

## 1 Introduction

Venous varix, also known as venous aneurysm or ectasia, is a rather common accompanying intracranial lesion in patients with brain arteriovenous malformation (BAVM). It is defined as a vessel at least two times the diameter of the draining vein.^[1]^ The presence of multiple feeders causing high flow shunt to the draining veins and steno-occlusive venopathy along the distal end of the AVM draining venous system are regarded as the pathophysiological bases for the formation and rupture of venous varix.^[2]^

Generally, although prone to rupture, a venous varix of BAVM is always asymptomatic, and no special management targeting the varix is warranted.^[3]^ However, when it becomes symptomatic (e.g., exhibits bleeding, compresses the brain parenchyma, or causes obstructive hydrocephalus or headache), treatment targeting the varix is needed.[^2^,^4^]

In this report, we present a case admitted for hemiparesis caused by a BAVM varix. After partial transarterial embolization (TAE) of the BAVM nidus, the venous varix spontaneously regressed. A literature review of similar reports was also performed.

## 2 Case report

An 11-year-old girl was admitted complaining of left hemiparesis for 4 days. She was previously healthy and denied any history of similar ictus. She was alert, and a physical examination performed upon admission was unremarkable except for the left hemiparesis.

**Figure 1:**
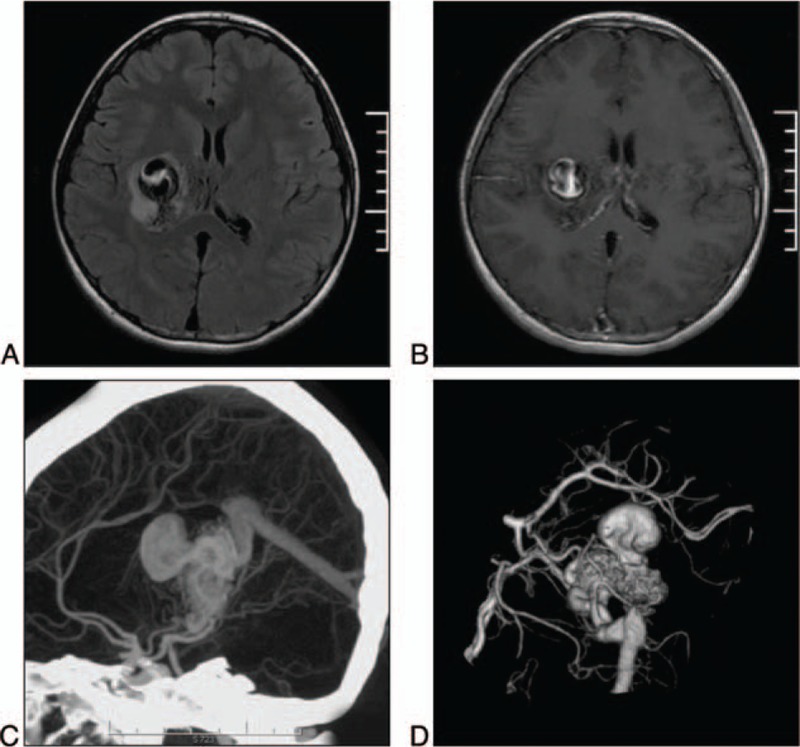
A: The FLAIR sequence on MRI shows a linear and round flow void and perilesional edema in the region of the right basal ganglia, indicating a BAVM. A signal with mixed intensity is noted in the round lesion, in accordance with the formation of an intraluminal thrombosis. B: Gadolinium-enhanced T1 sequence shows peripheral enhancement of the round lesion. C-D: MIP and 3-D CTA showing that the BAVM is fed by the ipsilateral PCA and AchA and drains into the vein of Galen. A large varix is also noted at the top of the BAVM. AchA = anterior choroidal artery, BAVM = brain arteriovenous malformation, CTA = computed tomography angiography, FLAIR = fluid attenuation inversion recovery, MIP = maximum intensity projection, PCA = posterior cerebral artery.

Head magnetic resonance imaging (MRI) showed a linear and round flow void and perilesional edema in the region of the right basal ganglia (Fig. 1A), indicating a BAVM (3.0 cm maximal diameter). A signal with mixed intensity is noted in the round lesion, in accordance with the formation of intraluminal thrombosis. Gadolinium-enhanced MRI showed peripheral enhancement of the round lesion (Fig. 1B).

Computed tomography angiography (CTA) showed that the BAVM was fed by the ipsilateral posterior cerebral artery (PCA) and anterior choroidal artery (AchA) and drained into the vein of Galen (Fig. 1C-D). A large varix (1.5 cm diameter) was also noted at the top of the BAVM and was consistent with the round flow void observed at the right basal ganglia on MRI. The Spetzler-Martin grading scale was grade IV.

**Figure 2:**
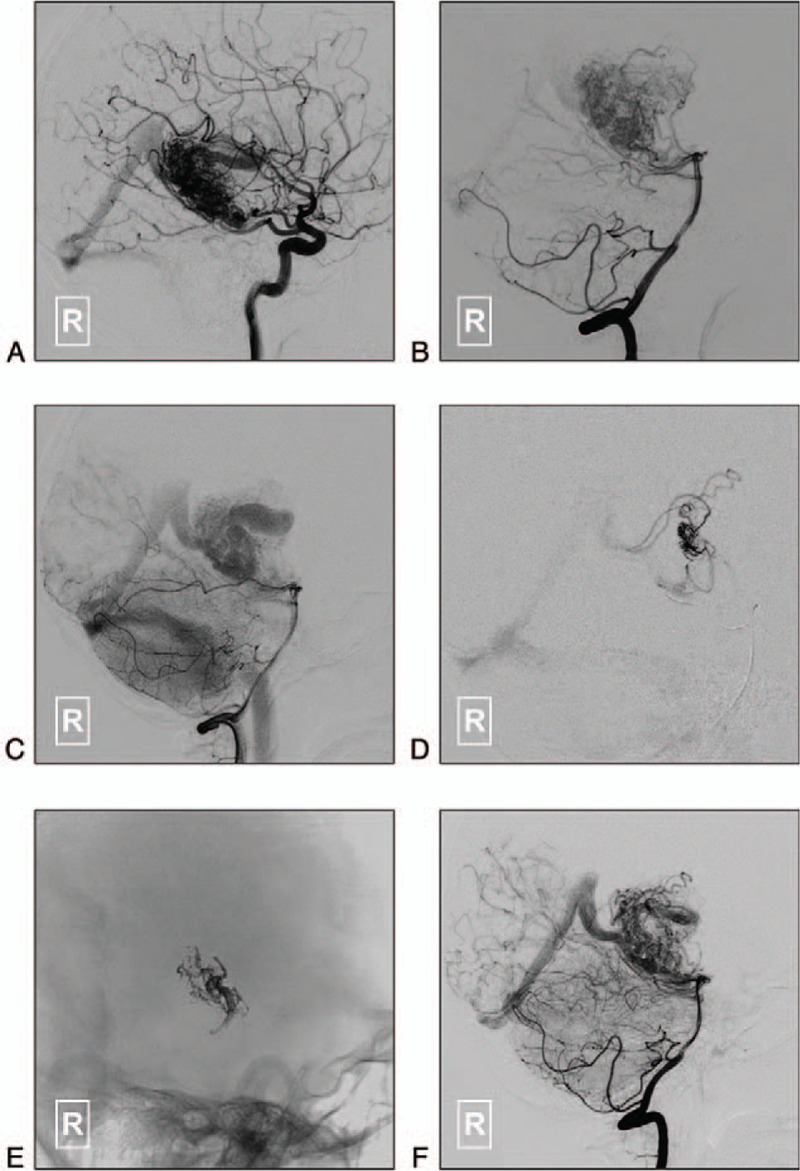
A-B: Lateral view of the right ICA (A) and VA (B) on DSA shows that the BAVM is supplied by branches of the right AchA and PCA and perforators of the thalamus. C: A later phase of the right VA angiography shows the BAVM draining into the vein of Galen through 2 draining paths. The superior draining vein is exceedingly enlarged and forms a varix. The inferior draining vein is also tortuous and dilated. D: Superselective angiography shows the main compartment of the BAVM nidus. E: Onyx casting in the BAVM nidus. F: The blood flow to the varix was clearly decrease after Onyx injection. AchA = anterior choroidal artery, BAVM = brain arteriovenous malformation, DSA = digital subtraction angiography, ICA = internal carotid artery, PCA = posterior cerebral artery, VA = vertebral artery.

As her symptoms of hemiparesis were suspected to be caused by the mass effect of the varix, a TAE of the BAVM feeders under general anesthesia was planned. Intraprocedural digital subtraction angiography (DSA) of the right internal carotid artery (ICA) and vertebral artery (VA) showed that the AVM was fed by branches of the right AchA and PCA and perforators of the thalamus (Fig. 2A-B). There were 2 draining veins. The superior draining vein was exceedingly enlarged and formed a varix. The inferior draining vein was also tortuous and dilated (Fig. 2C).

A 6F guiding catheter was positioned at the petrous portion of the right ICA. A Marathon microcatheter (Medtronic Neurovascular, Minneapolis, MN) was navigated to the feeding arteries of the BAVM to identify the wedged position of the microcatheter tip without any normal branches proximal to it. Then, the dimethyl sulfoxide (DMSO) syringe was directly attached to the hub of the microcatheter. The dead space of the microcatheter (0.3 mL) was filled with DMSO over 1 to 2 minute, under the guidance of a blank roadmap mask. The liquid embolic agent (Onyx 18, Medtronic Neurovascular, Minneapolis, MN) was slowly injected at a rate of approximately 0.16 mL/minute. The injection would be paused for 15 seconds if the Onyx refluxed along the catheter. Finally, partial casting of the BAVM was achieved. The blood flow to the varix was then clearly decreased (Fig. 2D–F).

**Figure 3:**
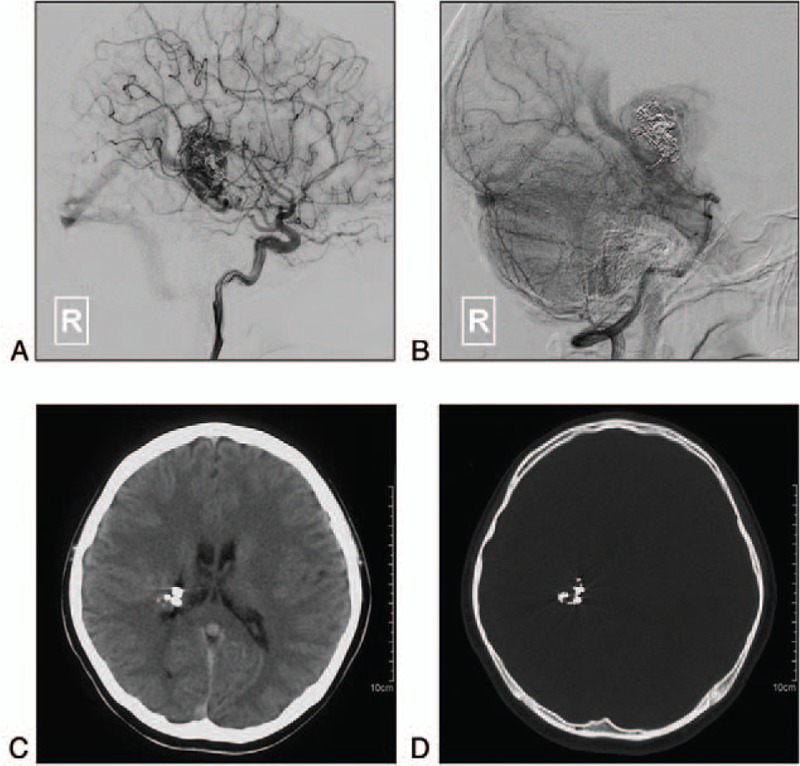
A-B: Follow-up DSA of the right ICA (A) and VA (B) 9 months later shows the residual BAVM nidus and the disappearance of the varix. C-D: CT shows the disappearance of the occupying lesion and perilesional edema. BAVM = brain arteriovenous malformation, CT = computed tomography, DSA = digital subtraction angiography, ICA = internal carotid artery, VA = vertebral artery.

The patient experienced a dramatic postprocedural recovery. The strength of her left limbs returned to normal in 1 month. A follow-up DSA performed 9 months later showed a decrease in the volume of the BAVM nidus and the disappearance of the varix (Fig. 3A-B). Computed tomography (CT) showed that the round lesion and perilesional edema at the right basal ganglia were invisible (Fig. 3C-D).

## 3 Ethics statement

Being a case report, our institution does not require formal Ethical Approval. Written informed consent was obtained from the patient and her parents for publication of this case report.

## 4 Discussion

There is still no global consensus regarding whether an unruptured BAVM should be treated.[^5^,^6^] Although BAVM-associated venous varix has long been regarded as a weak point and risk factor for future hemorrhage, hemorrhagic presentation of a varix is uncommonly reported.[^2^,^3^] When a BAVM varix ruptures or causes a mass effect, strategies targeting the AVM and/or varix are warranted.[^1^,^7^]

The aim of treatment could range from palliative to aggressive. For example, when a patient presents with hydrocephalus due to obstruction of the cerebrospinal fluid (CSF) circulation by a varix, palliative treatment could involve merely performing a CSF shunt without dealing with the BAVM and/or varix.^[4]^ In the case of aggressive treatment, strategies targeting the BAVM and/or varix are adopted. Although surgical resection is the most thorough and definite treatment for BAVM, it does carry some unpredictable risks, especially for high-grade BAVMs.

Hence, as illustrated by our case, surgical resection of the varix was difficult and dangerous due to its deep location and the eloquent nature of adjacent structures. Subtotal TAE of the BAVM nidus with no intervening of the varix was adopted. The patient experienced a favorable recovery and disappearance of the varix.

The using of Onyx for TAE can be accompanied with hemorrhagic and ischemic stroke, and equipment-related complications.^[8]^ Hemorrhagic stroke can be due to intraoperative rupture of the BAVM, too much liquid material injection into the nidus, or postembolization venous stagnation in and/or around the nidus.^[9]^ The causes of ischemic stroke can be arterial or venous, including antegrade and retrograde occlusion. Antegrade occlusion occurs when glue occludes a normal artery distal to the nidus. And retrograde occlusion occurs when glue refluxes to occlude normal arteries.^[10]^ The perforation of arterial feeders may occur as a result of wire access through small tortuous pial arteries.^[11]^

To further investigate the efficacy of pure TAE of the BAVM nidus in relieving the symptoms caused by a venous varix, we performed a literature search in PubMed. Five studies reporting 6 patients were identified, including the 1 case described in our report (Table 1).[^1^,^12^,^13^,^14^] Their ages ranged from 11 to 69 years old (47 ± 20.67 years old). Four patients (66.7%) experienced disappearance of the varices following partial or complete TAE of the BAVM feeders. One patient (16.7%) was reported to have decreased venous blood flow and no further progress. Only 1 (16.7%) patient experienced enlargement of the varix, which disappeared after subsequent resection of the BAVM nidus. All of the patients experienced a favorable recovery.

**Table 1:**
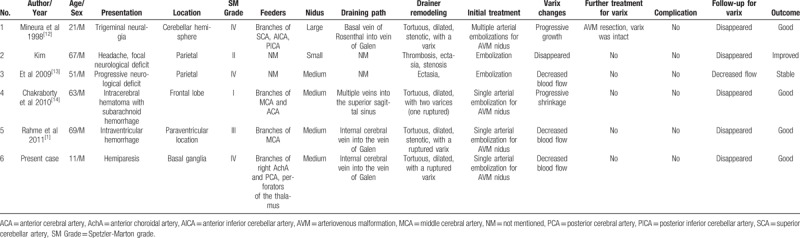
Clinical data of patients with transarterial embolization of the AVM nidus with a symptomatic venous varix.

The presence of high pressure arterial blood flow and steno-occlusive alteration of the draining venous system are the pathophysiological bases underlying the formation of a venous varix.[^1^,^2^] As a result of the difficulty and frequent lack of availability of a transvenous route, TAE of BAVM feeders is a theoretical treatment option. However, its efficacy has not been extensively explored. Some sporadic reports have achieved promising results using pure TAE.

The underlying therapeutic mechanism might be secondary thrombosis of the varices after TAE and the subsequent decrease in the blood flow of the BAVM. Hence, in the case of a BAVM-associated symptomatic varix, if surgical resection cannot be readily carried out, initial transarterial embolization of the feeders could be tried.

## Author contributions

**Conceptualization:** Guangming Wang, Kun Hou, Jinlu Yu.

**Data curation:** Guichen Li.

**Project administration:** Guangming Wang.

**Supervision:** Jinlu Yu.

**Writing – original draft:** Guichen Li, Jing Yu.

**Writing – review & editing:** Kun Hou, Jinlu Yu.
